# The Effect of Cognitive Reappraisal on Reactive Aggression: An fMRI Study

**DOI:** 10.3389/fpsyg.2018.01903

**Published:** 2018-10-15

**Authors:** Qi Jiang, Lulu Hou, Huanzhen Wang, Changran Li

**Affiliations:** ^1^Institution of Mental Health Education, Faculty of Psychology, Southwest University, Chongqing, China; ^2^Department of Psychology, School of Social and Behavioral Sciences, Nanjing University, Nanjing, China

**Keywords:** reactive aggression, cognitive reappraisal, provocative condition, TAP, fMRI

## Abstract

A number of empirical researches have shown that reactive aggression, which is the behavior that is impulsive, thoughtless, driven by anger, and causes harm toward another individual, can lead to a series of negative effects. Cognitive reappraisal may have the potential to reduce reactive aggression, but evidence for this effect in healthy populations is lacking. We randomly assigned participants to a Reappraisal Group (*n* = 19) or Control Group (*n* = 20) in a functional magnetic resonance imaging (fMRI) version of the well-established Taylor Aggression Paradigm (TAP). TAP was employed to elicit and measure reactive aggression, during which participants were informed that they would play a competitive reaction time task against two opponents in turn and the winner would punish the loser. The TAP used in this study separates the decision-making (during which participants were asked to set a punishment level for the opponent) and affective processes (during which the punishment was applied or received) that underlie reactive aggression. Behavioral data showed that there was no difference between the Reappraisal Group and Control Group in the punishment level selections (i.e., reactive aggression). However, on the neural level, cognitive reappraisal reduced the activation of left insula, right cuneus, and right middle frontal gyrus (MFG) during the decision phase, independently of the level of provocation. In addition, cognitive reappraisal reduced the activation of the caudate under the provocative condition when making decisions about aggressive behavior. The results of the outcome phase showed that, after winning a competition, cognitive reappraisal increased the activation of the right orbital middle frontal gyrus (OMFG) under the provocative condition and reduced the activation of the bilateral supplementary motor area (SMA) under the non-provocative condition. The results suggest that cognitive reappraisal would be effective in modulating the neural activity of reactive aggression.

## Introduction

Reactive aggression causes harm for both the individuals who perform the aggressive behavior and the individuals who suffer from the aggressive behavior. As such, its control has garnered theoretical and practical attention (Parke and Slaby, 1983). For example, the General Aggression Model (GAM) posits that personal and situational factors predict aggression via the mediating effect of internal state (i.e., cognition, emotion, and general arousal), and that controlled and effortful appraisal will lead to thoughtful behavior rather than aggressive behavior (Anderson and Bushman, 2002). Other theories have also suggested that cognitive reappraisal seems to be an effective method for reducing aggression by changing the hostile interpretation of the situation (e.g., Integrative Cognition Model, ICM; Wilkowski and Robinson, 2008). Nevertheless, the empirical evidence for these theories is lacking. Furthermore, although cognitive reappraisal is one of the components of cognitive behavioral therapy for the treatment of aggression in violent criminals (Wu, 2008), few experimental studies have focused on the aggression-reducing effect of cognitive reappraisal, especially in healthy populations.

Some researchers have tried to examine the relationship between cognitive reappraisal and reactive aggression via the provision of mitigating information. Indeed, lower levels of aggressive behavior have been found when the mitigating information was given (e.g., an apology from the provocateur or a justified explanation of the provocation; Ohbuchi et al., 1989; Dill and Anderson, 1995). From this, researchers have deduced that cognitive reappraisal could effectively reduce individuals’ aggressive behavior (Denson, 2015). However, other researchers have found that the provision of mitigating information failed to reduce aggression and even increased it (Zechmeister et al., 2004; Pedersen, 2006). The discrepancies between those studies were partly due to the differences of instructions (i.e., mitigating information). To this end, we used standard instructions from the emotion regulation field to examine the reactive aggression-reducing effect of cognitive reappraisal.

Furthermore, the neural processing underlying cognitive reappraisal is closely associated with reduced emotion response (Ochsner et al., 2002). Buhle et al. (2014) performed a meta-analysis of 48 neuroimaging studies of cognitive reappraisal and found that reappraisal consistently activated the prefrontal cortex, which plays an important role in controlling reactive aggression, and modulated the bilateral amygdala, which is involved in aggressive impulses. Indeed, the prefrontal region has been associated with reactive aggression. The classic case of Phineas Gage and other similar cases, whose prefrontal cortex were injured by accident subsequently became irritable and aggressive illustrate this (Damasio et al., 1994; Grafman et al., 1996). Moreover, animal studies have demonstrated that the amygdala plays a central role in a circuit that subserves the rapid detection of threats and the initiation of responses (Adamec, 1991; Ledoux, 2000; Maren, 2001). Furthermore, in humans, ablation of the amygdala via lesions or amygdalectomy and has been used to reduce aggression (Narabayashi et al., 1963; Lee et al., 1998). However, no studies have directly investigated the neural mechanism of the reactive aggression-reducing effect of cognitive reappraisal in normal humans. Additionally, these studies focused on animal and human lesion evidence have failed to capture the psychological characteristics after stimulus presentation (e.g., provocation). Recently, researchers modified the Taylor Aggression Paradigm (TAP; Taylor, 1967) to investigate the neural mechanism underlying reactive aggression (Krämer et al., 2007), which is useful for the present study.

In the variant of the TAP, each trial is divided into two phases. In the decision phase, the participant has to select a punishment level for the opponent in that trial, and in the outcome phase, the participant is informed whether he won or lost in the competition. In addition, the researchers set up two opponents (i.e., one non-provocative and one provocative) to study the effect of provocation on reactive aggression. In a prior study conducted by Krämer et al. (2007), contrasting the provocative against the non-provocative condition in the decision phases yielded activation of the rostral and dorsal ACC and the anterior insula, indicating that provocation by an unfair opponent probably elicited negative emotions and higher arousal. Contrasting feedback of winning against that of losing in the outcome phase yielded activation of the ventral striatum, suggesting that winning the competition is perceived as rewarding (Krämer et al., 2007). In another study, combining fMRI and the TAP, researchers found that the medial prefrontal cortex (mPFC) was activated during the decision phase, which was positively associated with the punishment level selected. The authors argued that activity in the mPFC likely reflected regulation of emotional conflict (Lotze et al., 2007). There are also other studies used the TAP to investigate the neural mechanism underlying the relationship between individual differences (e.g., emotional reactivity to threat or serotonin level) or situational factors (e.g., anger facial expressions of opponents) and reactive aggression (Krämer et al., 2011; Beyer et al., 2014, 2015). Therefore, this paradigm would be effective in capturing the differences in psychological processes underlying reactive aggression due to different personal and situational factors. However, there have been no studies investigating the aggression-reducing effect of cognitive reappraisal by this established paradigm.

Above all, in the present study, we firstly used an fMRI version of the well-established TAP to explore the behavior and neural effect of cognitive reappraisal on reactive aggression. In the behavior level, we assumed that cognitive reappraisal would reduce reactive aggression. In other words, we hypothesized that the participants who received cognitive reappraisal instruction would select lower levels of noise punishment for their opponents than those in the Control Group. On the neural level, we expected that cognitive reappraisal increased activation in the prefrontal cortex, especially in the mPFC, and reduced activation in the amygdala.

## Materials and Methods

### Participants

Forty-three undergraduates (20 males, aged 19–22 years, mean 20.35 ± 0.88 years; 23 females, aged 18–21 years, mean 19.74 ± 0.81 years) were recruited from Southwest University in China. All participants were right-handed and free of any psychiatric or neurological disorder. Following the procedures used in previous studies (Beyer et al., 2014, 2015), four participants were excluded from data analysis due to excessive movement (>4 mm) during functional scanning. Therefore, 39 participants (19 females, mean age = 20.1 ± 0.9 years) were ultimately included in the analyses and randomly assigned to a Reappraisal Group (*n* = 20) or Control Group (*n* = 19). This study was approved by the ethics committee of Southwest University. Furthermore, all participants gave their written informed consent upon arrival at the laboratory. After the experiment, each participant was paid 50 Yuan for participation.

### Materials

#### TAP

**FIGURE 1:**
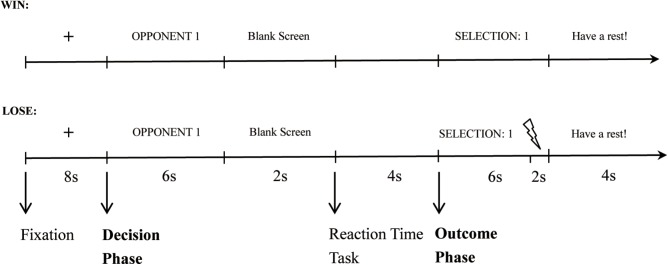
The time course for a single win and loss trial, respectively.

Participants were instructed that they would be playing successive competitive reaction-time trials against one of two opponents in turn inside the laboratory. At the beginning of each trial, a fixation cross was presented for 8 s. Then participants were shown the opponent for the coming competition (“Opponent 1” or “Opponent 2”). During this time, the participant had to select the punishment level (decision phase). The white noise levels ranged from 1 (70 dB) to 8 (105 dB) in 5-dB increments. During the practice stage, participants heard all of the noise levels. After a 2 s blank screen, the reaction-time task commenced, during which participants had to press a button as quickly as possible upon the appearance of a white circle (competition phase). Next, the participant was informed whether she/he won or lost and the selection of the opponent (outcome phase). In trials where the participant lost, the punishment noise was presented for 2 s, while in trials where they won there was a 2 s delay of the feedback screen. That is, in both the loss and win trials, the feedback screen was presented for 6 s, and it was accompanied by white noise in the last 2 s in the loss trials but not in the win trials. At the end of each trial, participants had 4 s to rest before beginning the next trial. **Figure 1** shows the time course for a single win and a single loss trial, respectively.

### Procedure

Upon completion of the questionnaires, participants completed 60 trials of the TAP to familiarize themselves with the experimental procedure. Then they were randomly assigned to the Reappraisal Group or Control Group. The instructions given to the Reappraisal Group were adapted from an instruction used by Denson et al. (2011), which emphasized the adoption of a neutral attitude toward the probably provocative situation. The instructions were as follows: “The second stage is the formal experimental stage. The procedure for the formal experiment is same as that of the practice stage, and you are required to compete against two opponents 60 times (i.e., 30 times each). Please pay attention to the fact that you can either win or lose each competition and if you lose the competition, you will receive low or high levels of punishment. In addition to your performance in the task, however, we would like to assess your emotion regulation ability. Therefore, it is very important that you try to adopt a neutral or objective attitude toward the outcomes and the punishment levels that your opponents set for you. To do so, try to think about the punishment levels that your opponent has set for you as random, rather than as personal or in any way emotionally relevant to you. Perform the task carefully, but please try to think about your opponent’s selections of in such a way that allows you to maintain a neutral mood. Once you have read and understood the above instructions, inform the lab assistant to start the formal experiment.” Participants in the Control Group received the following instructions: “The second stage is the formal experimental stage. The procedure for the formal experiment is same as that of the practice stage, and you are required to compete against two opponents in 60 times (i.e., 30 times each). Once you have read and understood the above instructions, inform the lab assistant to start the formal experiment.”

The formal experiment was divided into three blocks with 20 trials per block. In each block, participants completed 10 trials for each opponent in pseudo randomized order, and won 50% of the trials. To keep the social interaction plausible, we refer to the setting of Beyer et al. (2015). That is, the trials in which participants lost against each opponent were distributed unequally across the three blocks, as follows: seven trials lost against Opponent 1 and three trials lost against Opponent 2 in the first block, five trials lost against Opponent 1 and five trials lost against Opponent 2 in the second block, and three trials lost against Opponent 1 and seven trials lost against Opponent 2 in the third block. The outcomes of the reaction time task and the opponents’ punishment selections were controlled by the computer. Opponent 1 selected mainly low punishment levels ranging from Level 1–4 (*M* = 2.0), while Opponent 2 selected mainly high punishment levels ranging from Level 5–8 (*M* = 6.5).

### Questionnaires and Rating Scales

To ensure the equivalence of personality and affective state between the two groups, we assessed participants’ trait aggression, cognitive reappraisal, and positive and negative affect. The trait aggression was assessed with the Aggression Questionnaire (AQ; Buss and Perry, 1992), which included four subscales: physical aggression, verbal aggression, anger, and hostility. Items of the physical aggression (nine items) and verbal aggression (five items) scales ask about the hurting or harming of others, which represents motor component of aggression. Items of the anger scale (seven items) ask about the physiological arousal and preparation for aggression, which represents the emotional or affective component of behavior. The hostility scale (eight items) measures the feelings of ill will and injustice, which represents the cognitive component of aggressive behavior. Participants rated statements on 5-point scale from 1 (extremely uncharacteristic of me) to 7 (extremely characteristic of me) to indicate how much the behavior was like them. The AQ had an acceptable Cronbach’s alpha of 0.91 in the present study.

The Cognitive Reappraisal subscale of the Emotion Regulation Questionnaire (ERQ; Gross and John, 2003), translated into Chinese (Shi, 2012), was used to measure individual differences in habitual cognitive reappraisal. The scale consists of six items. Participants rated statements on a 7-point scale from 1 (strongly disagree) to 7 (strongly agree) to indicate how much the items were representative of their behavior. The scale had an acceptable Cronbach’s alpha of 0.80 in the present study.

The Positive and Negative Affect Scale (PANAS; Watson et al., 1988), translated into Chinese (Huang et al., 2003), was used to measure participants’ affective state in the preceding week. The scale consists of 20 items and 2 sub-dimensions: positive affect and negative affect. Participants rated statements on a 5-point scale from 1 (not at all) to 5 (very much) to indicate how strongly a statement applied to them. In the present study, the scale had acceptable Cronbach’s alphas of 0.88 and 0.89 for the positive affect subscale and negative affect subscale, respectively.

### fMRI Recording

A 3-T Siemens Trio MRI scanner (Siemens Medical Erlangen, Germany) was used to collect all images of the high-resolution T1-weighted brain structure acquired using a magnetization-prepared rapid gradient echo (MPRAGE) sequence (TR = 1,900 ms, TE = 2.52 ms, T1 = 900 ms, FA = 9°, 256 × 256 matrix, 176 slices, 1.00 mm slice thickness, voxel size = 1 mm × 1 mm × 1 mm) and T2^∗^-weighted images recorded using an echo-planar imaging (EPI) sequence [TR = 2,000 ms; TE = 30 ms; flip angle = 90°; field of view (FOV) = 220 mm × 220 mm; matrix size = 64 × 64; 32 interleaved 3 mm thick slices; in-plane resolution = 3.4 mm × 3.4 mm; interslice skip = 0.99 mm; volume = 320 per run]. As our stimulus was a noise, participants wore noise-canceling headphones (OptoACTIVE Active Noise Control) during the scanning.

### Data Analysis

Firstly, we used independent-samples *t*-tests and chi-square test to test the differences in personality, demographic variables, and emotion state of the two groups. For the behavioral data, in the decision phase, we conducted a 2 (provocative condition: Opponent 1, Opponent 2) × 2 (instruction style: Control Group, Reappraisal Group) ANOVA with participations’ selection as the dependent variable. To analyze differences in the time needed for the selection of the punishment level and the RTs in the competition phase, we conducted a 2 (provocative condition: Opponent 1, Opponent 2) × 2 (instruction style: Control Group, Reappraisal Group) ANOVA with participations’ RTs in the decision phase and competition phase as the dependent variables, respectively. Since overall RTs varied greatly between participants, we used z-standardized RTs in order to ensure comparability of the RT-differences under the different conditions.

For analysis of the MRI data, we used a toolbox for Data Processing Assistant for Resting-State fMRI (DPARSFA^1^) based on SPM8^2^ (Welcome Department of Cognitive Neurology, United Kingdom), which was run on the MATLAB R20011a software (Math Works Inc.)^3^. The preprocessing steps were as follows: (1) Images from the first 16 volumes at the beginning were discarded, because the noise-canceling headphones were starting up and there was no task for the participants during this time; (2) the remaining 302 images were corrected for slice timing and head motion correction to adjust the time series of the images; (3) the structural images were coregistered to the mean functional image and were subsequently segmented as gray matter, white matter and cerebrospinal fluid employing the new segment method; (4) each functional image was normalized to the standard Montreal Neurological Institute (MNI) space with the application of DARTEL (diffeomorphic anatomical registration through exponentiated lie algebra); (5) after normalization, spatial smoothing was performed with a 6 mm full-width-half-maximum Gaussian kernel.

We estimated a first-level General Linear Model (GLM) per subject for the decision phases and the outcome phase. To model the decision phases, the two opponents and motion regressors are included in the design matrix. In addition, in order to model the outcome phases, the two opponents, two outcomes, and motion regressors are included in the design matrix. On the second level, we estimated a flexible factorial design with three factors (subject, instruction type, and provocative condition) in the decision phase. For the outcome phase, we contrasted win > lose and lose > win first and then estimated a flexible factorial design with three factors (subject, instruction type, and provocative condition) for win and lose trials separately. Subject factors were not included in the design matrices. It is important to note that, referring to prior studies (e.g., Beyer et al., 2014), two kinds of different multiple comparisons correction are often used for different contrasts. Specifically, for the main effects of within-subject variables, we set the voxel-level statistical threshold at *p* < 0.001 and corrected with a cluster-level of *p* < 0.05 at the family-wise error (FWE). For the main effect of between-subject variable and interaction effects involving between-subject variable, we set the voxel-level statistical threshold at *p* < 0.001 and corrected with cluster-level of *p* < 0.05 at the non-stationarity (ns), unless restated.

## Results

### Questionnaire and Rating Scales

The results indicated that there were no group differences in terms of age (Control Group: 20.00 ± 0.75; Reappraisal Group: 20.15 ± 0.99, *t_37_* = −0.53, *p* > 0.05) or gender (Control Group: 9 females and 10 males; Reappraisal Group: 10 females and 10 males, *χ*^2^(1) = −0.02, *p* > 0.05).

The mean AQ score was 70.23 (*SD* = 16.84) and was not different between the two groups (Control Group: 73.30 ± 17.15; Reappraisal Group: 67.31 ± 16.48; *t*_37_ = 1.11, *p* > 0.05). The mean score of cognitive reappraisal was 30.38 (*SD* = 5.58) and there were no differences between the two groups (Control Group: 29.31 ± 4.83; Reappraisal Group: 31.39 ± 6.16; *t*_37_ = −1.17, *p* > 0.05). The mean score of positive affect was 30.15 (*SD* = 7.01) and there were no differences between the two groups (Control Group: 29.21 ± 7.60; Reappraisal Group: 31.05 ± 6.47; *t*_37_ = −0.82, *p* > 0.05). The mean score of negative affect was 17.92 (*SD* = 5.79) and there were no differences between the two groups (Control Group: 18.00 ± 5.71; Reappraisal Group: 17.85 ± 6.01; *t*_37_ = 0.08, *p* > 0.05).

### Behavioral Data

**FIGURE 2:**
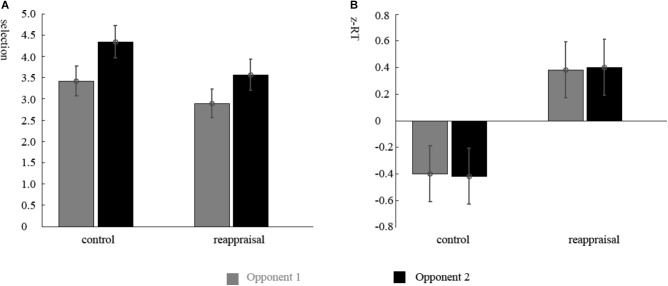
Behavior results. **(A)** Mean punishment levels selected in the decision phase and **(B)** mean standardized RTs in the competition phase for participants in Reappraisal Group and Control Group under the non-provocative and provocative conditions.

The ANOVA of the participants’ selections showed that there was a significant main effect of the provocative condition. Participants chose higher punishment levels for Opponent 2 than Opponent 1 [Opponent 1: 3.14 ± 1.53; Opponent 2: 3.94 ± 1.69; *F*(1,37) = 11.56, *p* < 0.01; **Figure 2A**]. The main effect of instruction type and the interactions were not significant (*ps* > 0.05).

The ANOVA of the RTs of the decision phase showed that there were no differences among conditions (*ps* > 0.05). The RTs of the competition phase showed that there was a significant main effect for group. The RTs of participants in the Reappraisal Group was greater than in Control Group [Control Group: 281.56 ± 93.97 ms; Reappraisal Group: 360.44 ± 82.43; *F*(1,37) = 7.81, *p* < 0.01; **Figure 2B**]. The other effects were not significant (*ps* > 0.05).

### fMRI Data

#### Decision Phase

**FIGURE 3:**
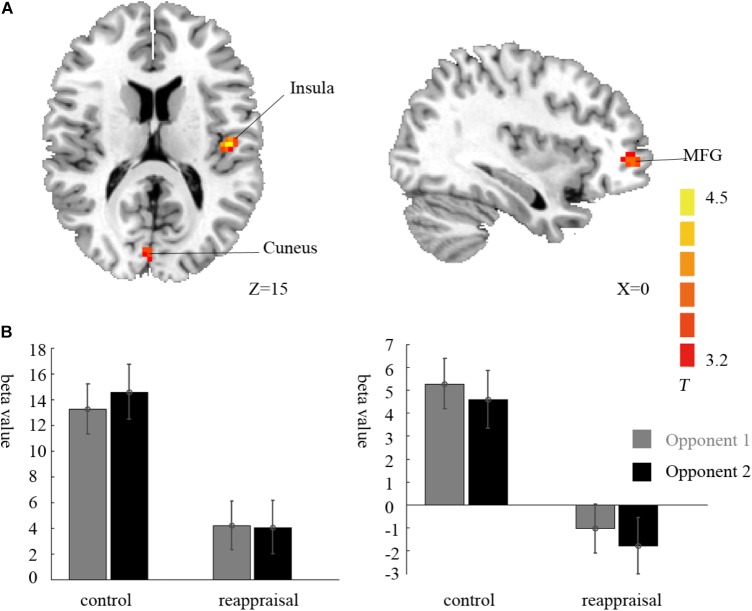
Imaging results for the decision phase. **(A)** Shows the main effect of instruction type with an decreased BOLD response in the right cuneus, left insula and left MFG (cluster-defining threshold *p* < 0.001, uncorrected for multiple comparisons) in the Reappraisal Group compared to that in the Control Group. **(B)** Depicted corresponding ROI beta values for the cuneus (left) and the insula (right).

Contrasting the provocative > non-provocative condition for the decision phase showed no activation at the chosen significance threshold. When we examined the main effect of the instruction type, there was no activation at the chosen significance threshold either. Importantly for our research question, we used a more liberal threshold of *p* < 0.001, uncorrected, and a clustering threshold of 20 voxels and found that the activation of the right middle frontal gyrus (MFG), right cuneus, and left insula was greater in the Control Group than in the Reappraisal Group (see **Table 1** and **Figure 3**).

**Table 1:** Neural effects for the decision phase.

Region	Hemisphere	MNI coordinates	voxels	Cluster-peak *t*-value
**Control Group > Reappraisal Group**				
Insula	L	−45, −18, 15	22	4.94
Middle frontal gyrus	R	36, 51, 0	27	4.06
Cuneus	R	3, −84, 15	23	3.77
**Interaction: provocation × instruction group**				
Caudate	L	−18, 30, −12	37	4.36

**FIGURE 4:**
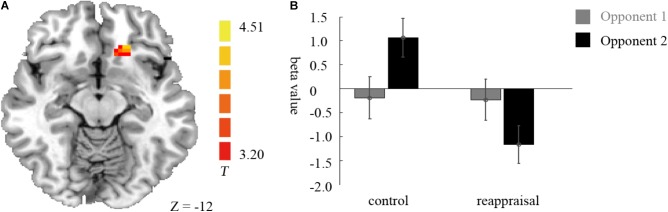
Imaging results for the decision phase. **(A)** Shows the interaction effect of provocative condition × instruction type with an decreased BOLD response in the left caudate (cluster-defining threshold *p* < 0.001, 0.05 ns-corrected at the cluster level) in the Reappraisal Group compared to that in the Control Group under the provocative condition only. **(B)** Depicted corresponding ROI beta values for the caudate.

Examining the interaction between provocative condition and instruction type, the left caudate was significantly activated at the chosen significance threshold (*p* < 0.001, ns correction). The average beta of the caudate was extracted from activation clusters for further analysis using REST (Song et al., 2011). The behavioral data results showed that for the caudate, the interaction between provocative condition and instruction type was significant [*F*(1,37) = 14.03, *p* < 0.01] and the simple effect test showed that cognitive reappraisal reduced the activation of the caudate significantly only under the provocative condition [*F*(1,37) = 15.89, *p* < 0.01], but not under the non-provocative condition [*F*(1,37) = 0.00, *p* > 0.10]. Additionally, for participants in the Control Group, the activation of the caudate was higher under the provocative condition compared to the non-provocative condition [*F*(1,37) = 8.92, *p* < 0.01], while for participants in the Reappraisal Group, the activation of the caudate was higher under the non-provocative condition compared to the provocative condition [*F*(1,37) = 5.30, *p* < 0.05]. In addition, the main effect of group was also significant [*F*(1,37) = 4.90, *p* < 0.05], and the activation of the caudate in the Control Group was higher than in the Reappraisal Group. However, the main effect of provocation was not significant [*F*(1,37) = 0.28, *p* > 0.10]. The results are displayed in **Table 1** and **Figure 4**.

#### Outcome Phase

Contrasting win against lose trials yielded significant activation of the bilateral middle temporal gyrus, left superior frontal gyrus, bilateral angular gyrus, left precuneus, bilateral postcentral gyrus, and left paracentral lobule. The opposite contrast showed activation in the bilateral superior temporal gyrus, left middle occipital gyrus, right cuneus, bilateral insula, left middle cingulate gyrus, bilateral SMA, and left operculum inferior frontal gyrus. The results are displayed in **Table 2**.

**Table 2:** Neural effects for the outcome phase.

Region	Hemisphere	MNI coordinates	voxels	Cluster-peak *t*-value
**Win > lose**				
Middle temporal gyrus	L	−60, −6, −24	333	7.69
	R	60, −39, −9	57	6.51
	R	63, −12, −21	104	7.23
	R	39, −63, −3	87	7.73
Superior frontal gyrus	L	−36, 21, 48	2458	10.09
Angular gyrus	L	−54, −60, 27	483	9.27
	R	51, −60, 30	313	8.20
Precuneus	L	6, −54, 30	379	8.67
Postcentral gyrus	L	−39, −36, 45	27	5.84
	R	30, −21, 60	103	6.82
Paracentral lobule	L	−6, −27, 63	141	7.28
**Lose > win**				
Superior temporal gyrus	L	−42, −33, 9	467	9.39
	R	51, −21, 3	375	10.26
Middle occipital gyrus	L	−15, −102, 9	74	8.01
Cuneus	R	21, −99, 9	90	8.80
Insula	L	−33, 15, 6	49	8.20
	R	36, 21, 3	39	7.73
Middle cingulate gyrus	L	−12, −27, 39	27	7.31
Supplementary motor area	L	−12, −3, 66	26	6.66
	R	−6, 12, 36	173	7.72
Operculum inferior frontal gyrus	L	−57, 6, 3	23	6.18

In the lose trials, there was no activation at the chosen significance threshold for the main effects of provocative condition and instruction type and their interaction. In the win trials, the interaction of provocative condition and instruction type showed significant right OMFG and bilateral SMA activation. Average beta values of these areas were extracted from activation clusters for further analysis.

**FIGURE 5:**
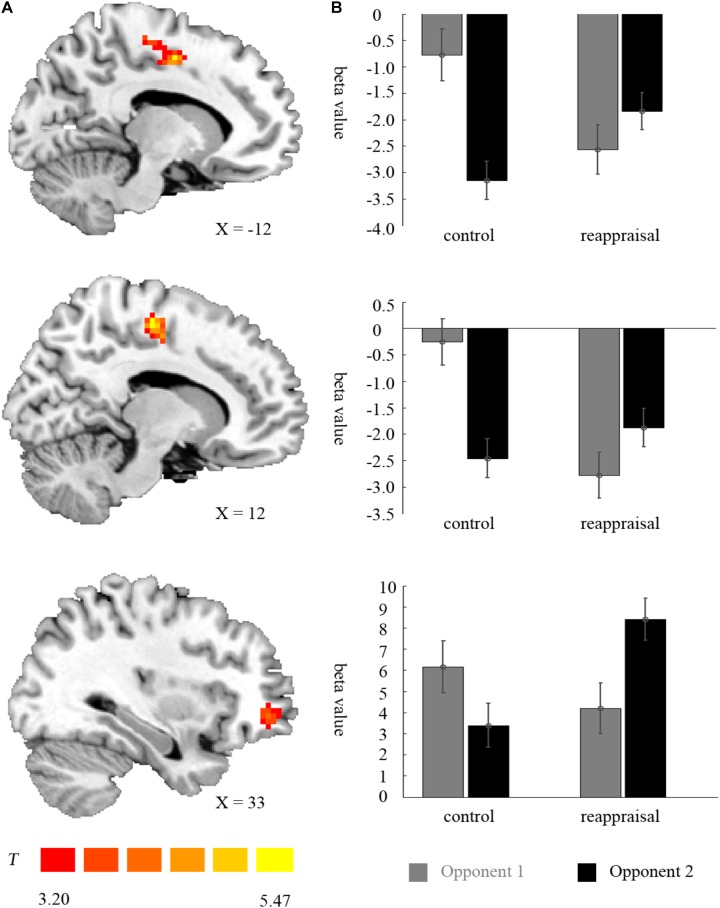
Imaging results for the outcome phase. **(A)** Shows the interaction effect of provocation condition × instruction type in the left SMA, right SMA, and right OMFG (cluster-defining threshold *p* < 0.001, 0.05 ns-corrected at the cluster level). **(B)** Depicted corresponding ROI beta values for the left SMA, right SMA, and right OMFG.

The behavioral data results showed that for the right OMFG, the interaction between provocative condition and instruction type was significant [*F*(1,37) = 18.34, *p* < 0.001]. The simple effect test showed that cognitive reappraisal increased the activation of the right OMFG significantly under the provocative situation [*F*(1,37) = 12.22, *p* < 0.01], but not under the non-provocative situation [*F*(1,37) = 0.52, *p* > 0.10]. Additionally, for participants in the Control Group, the activation of the right OMFG was higher under the non-provocative condition compared to the provocative condition [*F*(1,37) = 6.95, *p* < 0.05], while for participants in the Reappraisal Group, the activation of the right OMFG was higher under the provocative condition compared to the non-provocative condition [*F*(1,37) = 11.76, *p* < 0.01]. However, the main effects of provocative condition [*F*(1,37) = 0.26, *p* > 0.10] and instruction style [*F*(1,37) = 1.82, *p* > 0.10] were not significant. The results are displayed in **Table 3** and **Figure 5**.

**Table 3:** Neural effects for the outcome phase in the win trial.

Region	Hemisphere	MNI coordinates	voxels	Cluster-peak *t*-value
**Interaction: provocation × group**				
Supplementary motor area	R	12, −18, 57	56	5.93
	L	−12, −6, 48	59	5.62
Orbital middle frontal gyrus	R	33, 48, −6	59	4.19

For the left SMA, the interaction between provocative condition and instruction type were significant [*F*(1,37) = 26.25, *p* < 0.001] and the simple effect test showed that cognitive reappraisal reduced the activation of the left SMA under the non-provocative condition [*F*(1,37) = 6.85, *p* < 0.05], but increased the activation of the left SMA under the provocative situation [*F*(1,37) = 6.94, *p* < 0.05]. Additionally, for participants in the Control Group, the activation of the left SMA was higher under the non-provocative condition compared to the provocative condition [*F*(1,37) = 30.00, *p* < 0.001], while for participants in the Reappraisal Group, activation of the left SMA did not differ between conditions [*F*(1,37) = 2.96, *p* > 0.05]. In addition, the main effect of the provocative condition was also significant [*F*(1,37) = 7.41, *p* < 0.05] and activation of the left SMA under the non-provocative condition was higher than in the provocative condition. However, the main effect of instruction style was not significant [*F*(1,37) = 0.22, *p* > 0.10]. The results are displayed in **Table 3** and **Figure 5**.

For the right SMA, the interaction between provocative condition and instruction type was significant [*F*(1,37) = 36.22, *p* < 0.001] and the simple effect test showed that cognitive reappraisal reduced the activation of the right SMA under the non- provocative condition [*F*(1,37) = 16.68, *p* < 0.001], but not under the provocative condition [*F*(1,37) = 1.24, *p* > 0.10]. Additionally, for participants in the Control Group, the activation of right SMA was higher under the non-provocative condition compared to the provocative condition [*F*(1,37) = 35.67, *p* < 0.001], while for participants in the Reappraisal Group, the activation of the right SMA was higher under the provocative condition compared to the non-provocative condition [*F*(1,37) = 6.22, *p* < 0.05]. In addition, the main effect of provocative condition was also significant [*F*(1,37) = 6.43, *p* < 0.05] and the activation of the right SMA under the non-provocative condition was higher than in the provocative condition. However, the main effect of instruction style was not significant [*F*(1,37) = 3.65, *p* > 0.05]. The results are displayed in **Table 3** and **Figure 5**.

## Discussion

In the current study, we investigated the effect of cognitive reappraisal on reactive aggression on both the behavioral and neural level. On the behavior level, the present study does not support prior reports of a potential effect of cognitive reappraisal on reactive aggression during the decision phase. In addition, participants’ reactive aggression was higher when provoked than when not provoked. On the neural level, we found that cognitive reappraisal reduced the activation of the left insula, right cuneus, and right MFG during the decision phase, and reduced the activation of caudate under the provocative condition. The results of the outcome phase showed that after winning the competition, cognitive reappraisal was related to increased activation in the right OMFG under the provocative condition, and reduced activation of the bilateral SMA under the non-provocative condition.

### Decision Phase

Researchers have called reappraisal that occurs before the onset of the emotional response early reappraisal, while reappraisal occurring after a negative emotional experience is called late reappraisal (Sheppes and Gross, 2011). Many studies have focused on late reappraisal while only a few studies have focused on the aggression-reducing effect of cognitive reappraisal by providing mitigating information prior to the negative event (Fabiansson and Denson, 2012). However, early reappraisal is thought to require less mental effort than late reappraisal, thus requires more attention. In this study, we first used the instructions of cognitive reappraisal used in emotion studies to explore the reactive aggression-reducing effect of early cognitive reappraisal. However, inconsistent with prior studies, our results suggested that cognitive reappraisal had no direct influence on reactive aggression during the decision phase on the behavior level. However, on the neural level, we found that cognitive reappraisal reduced the activation of the left insula, right cuneus, and right MFG during the decision phase.

The cognitive reappraisal was related to a reduced BOLD response in the left insula during the decision phase, which was independent of the provocative condition. This result is consistent with prior studies, which found insula activation when cognitive reappraisal was used to regulate emotion processing (Goldin et al., 2008). The insula (especially the anterior insula) has been found to be involved in much of the processing, such as interoception, awareness of body movement, emotional awareness and so on (Craig, 2009). The insula is well situated for the integration of information relating to bodily states into higher-order cognitive and emotional processes, receiving information from the thalamus and sending output to a number of other limbic-related structures, such as the amygdala, the ventral striatum, and the orbitofrontal cortex, as well as to motor cortices (Craig, 2003). Tamietto et al. (2015) studied one patient with visual extinction following right parietal damage and found that the insula was a key brain region in the integration between peripheral arousal and the central mapping of ongoing visceral and sensory-motor changes, which was critical for conscious visual experience of emotional signals. Another study found the insula was relevant in decision-making under competition or unreciprocated cooperation (Rilling et al., 2008). Based on the above theories and research, we inferred that the decision situation was a kind of potential emotional signal; the reduction in the activity within the Reappraisal Group might reflect the reduction of conscious experience of emotional signals when making a decision about aggression. That is, for the Control Group, the decision process induced a stronger emotional experience than that did in the Reappraisal Group.

When making the decision whether perform an aggressive behavior, it is also important to understand the intent and ideas of the opponents. This consideration has been correlated with Theory of Mind (ToM). The activation of the MFG and cuneus may be related to ToM as suggested by previous neuroimaging studies (Mah et al., 2004). The dysfunction of these regions may lead to difficulties in social perception and making interpersonal judgments (Mah et al., 2004; Geraci et al., 2010). For example, in a prior study, participants were asked to complete a Stroop task in order to win either against a human-like competitor (human–human competition) or against a non-human competitor (human–machine competition). Contrasting the human-human competition (i.e., referred to ToM) with human-machine competition (i.e., did not refer to ToM), the cuneus, precuneus and MFG became activated (Polosan et al., 2011). In our study, besides the wish to reach an agreement with the opponents, participants wanted to understand the thoughts and feelings of opponents. This may have led to this neural activation in the Control Group relative to the Reappraisal Group, who was instructed to reinterpret the punishments in a neutral manner.

Cognitive reappraisal also reduced the activation of the caudate in the provocative condition, which is known as the reward area (Delgado et al., 2003; Hyman et al., 2006). Fischbacher et al. (2004) found that when participants were given the chance to punish the defector in a game, the caudate was activated. The activity intensity of the caudate was correlated with the amount of money participants were willing to spend on punishing the defector in their study. Krämer et al. (2007) found a positive relationship between caudate activity in the decision phase of the TAP and punishment level selected. Furthermore, Beyer et al. (2014) found that the contrast value for the caudate (provocative vs. non-provocative condition) during the decision phase was positively correlated with aggressive behavior, lending further support to a connection between caudate activation and reactive aggression. Our study found that caudate activation was not different between groups in the non-provocative condition, while there was a difference in the provocative condition. Our results indicated that when participants played against the non-provocative opponent, this predominant response (selecting high punishment levels) had to be suppressed in favor of a non-aggressive response. In the provocative condition, the participants in the Control Group tended to select high punishment as the predominant response while the participants who received the cognitive reappraisal instruction could suppress the automatic predominant response.

Contrary to our hypothesis, we found no effect of cognitive reappraisal on amygdala activity during the decision phase, which is similar to a previous study using the same paradigm, which found that amygdala activity was not activated in the provocative condition due to individual differences (Beyer et al., 2014). This is partly because the amygdala plays an important role in the generation of reactive aggression and previous studies have shown that the amygdala response habituates rapidly (Yang et al., 2009). However, we could not find changes in amygdala activity with the conditions in this paradigm as did the most of the studies that used the same paradigm (Krämer et al., 2011; Beyer et al., 2014). In addition, we did not find an effect of cognitive reappraisal on the mPFC as expected, which is consistent with a previous study with TAP (Krämer et al., 2011; Beyer et al., 2014). In fact, it has recently been argued that the mPFC is a crucial structure for the development of socially appropriate behavior rather than the inhibition of anger or aggression on a trial-wise basis (Blair, 2012). Moreover, the evidence of the role of the mPFC in the suppression of anger and aggression is mainly derived from studies with patients and violent criminals (Grafman et al., 1996), whose inhibitory control function is reduced, and whether it is also observed in healthy people should be considered in more studies.

### Outcome Phase

Contrasting winning against losing trials showed significant activation of the bilateral middle temporal gyrus, left superior frontal gyrus, bilateral angular gyrus, left precuneus, bilateral postcentral gyrus, and left paracentral lobule. This result replicates previous findings of increased precuneus activation in win trials, which plays an important role in ToM (Völlm et al., 2006; Geraci et al., 2010; Blair, 2012). Our study indicates that participants showed empathy for incoming opponents’ punishment. However, previous studies found increased activity in the caudate in win trials (Krämer et al., 2007; Beyer et al., 2014), which we could not replicate with the current study. Contrasting losing against winning trials showed activation in the bilateral superior temporal gyrus, left middle occipital gyrus, right cuneus, bilateral insula, left middle cingulate gyrus, bilateral SMA, and left operculum inferior frontal gyrus. This result is consistent with previous studies (Krämer et al., 2007; Beyer et al., 2014) and underlines the role of the insula in processing of emotions, especially aversive events (Krämer et al., 2007; Beyer et al., 2014).

Of interest for our research question, we found that in the win trials, the interaction between provocative condition and instruction type was related to bilateral SMA and right OMFG activation. The prefrontal cortex, especially the medial prefrontal cortex plays an important role in emotion regulation (Nitschke et al., 2006). Thus, our results indicate that, in the provocative condition, the outcome evoked strong emotions and participants in the Reappraisal Group employed more cognitive resources to regulate their emotional state during the outcome phase while participants in the Control Group could not. However, in the non-provocative condition, all participants could regulate weak emotion. Moreover, the SMA is a part of the pain matrix, which inhibits the skeletomotor impulses to avoid the stimulus in the context of painful information (Wager et al., 2008). In our study, bilateral SMA showed similar activation in the win trials. A previous study scanned whole brain activity when the participants viewed animated visual images and found that the left SMA was activated when contrasting animation that showed others in pain caused by accident versus others in no pain. Furthermore, participants who were diagnosed as having conduct disorder (CD) showed greater right SMA activation than healthy participants in this contrast (pain caused by accident vs. no pain). This result suggests that SMA activation was greater in participants with CD, who are frequently more aggressive than those without CD, in other words. This suggests that an individual whose inhibitory control ability is worse is more sensitive to the pain of others (Decety et al., 2009). Our results indicated that in the winning trials in the non-provocative condition, participants in the Control Group were more sensitive to the pain of others. However, in the provocative condition of winning trials, participants in the Control Group were angrier in response to the injustice than having enjoyment for the outcome. This was related to the reduction of bilateral SMA activation.

### Limitations

The study was subject to several limitations. First, though the white noise level was set up referring to prior studies (e.g., Watkins et al., 2015), the lack of standard ratings for the white noise in our study limits the explanatory power of the penalty of the white noise. Second, no other rating was given to measure the participants’ anger toward their opponents, which may have some effects on explaining the neural results.

## Conclusion

The present study does not support prior reports of a potential effect of cognitive reappraisal on reactive aggression during the decision phase on the behavior level. However, we found that on the neural level, cognitive reappraisal reduced activation in areas involved in cognitive processes (i.e., insula) and ToM (i.e., cuneus and MFG), and reduced the activation in areas involved in reward circuits (i.e., caudate) under the provocative condition when making decisions to engage in aggressive behavior. The results of the outcome phase show that after winning the competition, cognitive reappraisal is related to increases in the activation of the prefrontal cortex under the provocative condition, and reductions in the activation of the pain matrix (i.e., SMA) under the non-provocative condition. These results support the view that cognitive reappraisal would be effective in moderating the mental processes during reactive aggression on the neural level. Future studies should further examine the effect of cognitive reappraisal on reactive aggression. Such studies can be conducted by comparing the behavioral and neural differences among those scoring high and low on habitual cognitive reappraisal.

## Ethics Statement

All procedures performed in studies involving human participants were in accordance with the ethical standards of the institutional and/or national research committee and with the 1964 Helsinki Declaration and its later amendments or comparable ethical standards. Written informed consent was obtained after detailed explanation of the study protocol, which was approved by the Ethics Committee of Southwest University. The Institutional Review Board at Southwest University (SWU) in Chongqing, China approved this consent procedure. Written informed consent was obtained from all participants. The Institutional Review Board at SWU approved all procedures. Informed consent was obtained from all individual participants included in the study.

## Author Contributions

QJ and LH conceived and designed the experiments. LH, HW, and CL performed the experiments. LH analyzed the data. QJ and LH wrote the paper.

## Conflict of Interest Statement

The authors declare that the research was conducted in the absence of any commercial or financial relationships that could be construed as a potential conflict of interest.

## Footnotes

1 http://restfmri.net/forum/DPARSF

2 http://www.fil.ion.ucl.ac.uk/spm/

3 http://www.mathworks.com
